# The efficacy of adalimumab in psoriatic arthritis concomitant to overlapping primary biliary cholangitis and primary sclerosing cholangitis: a case report

**DOI:** 10.1186/s12891-016-1335-x

**Published:** 2016-11-22

**Authors:** Teresa Del Ross, Amelia Ruffatti, Annarosa Floreani, Ariela Hoxha, Leonardo Punzi

**Affiliations:** 1 Rheumatology Unit, Department of Medicine, University Hospital of Padua, Padua, Italy; 2 Department of Surgery, Oncology and Gastroenterology, University Hospital of Padua, Padua, Italy

**Keywords:** Psoriatic arthritis, Primary biliary cirrhosis, Primary sclerosing cholangitis, Anti-TNF-alpha drugs

## Abstract

**Background:**

The overlap syndrome of primary biliary cholangitis (formerly called primary biliary cirrhosis) and primary sclerosing cholangitis is an extremely rare condition that has never been described in association with other immune-mediated diseases, including psoriatic arthritis. While treatment with anti-Tumour Necrosis Factor-alpha (TNF-α) agents has proved to be effective in inflammatory arthropathies such as psoriatic arthritis, they have been employed in only a limited number of patients with autoimmune hepatitis, and their effectiveness is unclear.

**Case presentation:**

We report the case of a 51-year-old female affected with psoriatic arthritis concomitant to overlapping primary biliary cholangitis and primary sclerosing cholangitis in whom 28 months of adalimumab treatment improved the symptoms of the inflammatory arthropathy as well as those of both cholangiopathies.

**Conclusion:**

Our results suggest that further studies examining the therapeutic role of this particular TNF-α blocker are warranted in cholestatic autoimmune hepatitis patients, and in particular in those individuals in whom the disease is associated with inflammatory arthropathies.

## Background

While a variety of immunological diseases, including rheumatoid arthritis (RA), have been reported in patients affected by primary biliary cholangitis (formerly called primary biliary cirrhosis) (PBC) [1], primary sclerosing cholangitis (PSC) is strongly associated with inflammatory bowel disease (IBD), and particularly with ulcerative colitis (UC) [2]. Until now only one case of a psoriatic arthritis (PsA)-PBC association has been reported [3], and, to the best of our knowledge, no cases of PsA associated to PSC have ever been described in the literature. The PBC-PSC overlap syndrome is, moreover, an extremely rare condition that has been described in only eight cases [4].

The development of Tumour Necrosis Factor-α (TNF-α) blockers has represented a milestone in the treatment of inflammatory joint diseases. Adalimumab, which is a fully human anti-TNF-α monoclonal antibody, has been shown to be effective in achieving remission and in preventing radiographic progression of joint damage in patients with RA and other inflammatory arthropathies, including PsA. It has also been found to be efficacious in treating skin and nail lesions in PsA patients [5]. Although a pathogenic hypothesis [6, 7] would justify the use of anti-TNF-α drugs for both PBC and PSC, treatment outcome is nevertheless uncertain. A double-blind, placebo-controlled, randomized study examining Infliximab treatment in PSC patients concluded that it was inefficacious in the small group studied [8]. It was likewise ineffective in a case of PBC associated with RA [9], although it did prove to be efficacious in a case of PSC that was complicated by ankylosing spondylitis and UC [10]. Etanercept led to an improvement in both liver enzymes and arthritis in two cases of RA associated with PBC [9, 11]. Prescribed to treat IBD in a patient who was also suffering from PBC, Adalimumab treatment led to a favourable clinical and laboratory response with regard to both disorders [12]. To date, no cases of PBC, PSC, or overlapping PBC-PSC associated with inflammatory arthropathies have been treated with Adalimumab. The current report describes a case in which Adalimumab treatment led to clinical improvement in arthritis symptoms and in nail lesions and also lowered cholestasis indices in a PsA patient with overlapping PBC-PSC syndrome.

## Case presentation

In September 2011 a 51-year-old Caucasian woman with a history of psoriasis and type-2 diabetes mellitus developed a mild psoriasis together with dactylitis affecting the right fourth finger and arthritis of the fourth metacarpophalangeal (MCP) joint of the right hand. In November 2011 she was referred our outpatient clinic. Dactylitis of the fourth finger of the right hand and swelling, redness and pain of the distal interphalangeal (DIP) joint of the second digit of the right hand were noted during the physical examination. Laboratory results at that time included: Erythrocyte sedimentation rate (ESR) = 100 mm/h (normal range 0–39 mm/h), C-reactive protein (CRP) = 3.99 mg/l (normal range 0–6 mg/l), gamma-glutamyltransferase (γGT) = 237 IU/l (normal range 3–45 IU/ml), and alkaline phosphatase (ALP) = 201 IU/l (normal range 53–141 IU/ml). The patient was treated with methylprednisolone for 6 months; treatment was started with doses of 16 mg/die that were tapered to 4 mg/die. She also received 15 mg weekly oral Methotrexate (MTX) for 6 months beginning in May 2012 with poor results. In March 2012, hand X-rays uncovered periostitis of the proximal phalanx of the right fourth digit; ultrasound (US) and powerdoppler evaluation revealed flexor tenosynovitis of the fourth finger of the right hand and a small erosion in the head of the fourth MCF of the right hand. An US-guided corticosteroid injection of the tendon sheath was adminstered.

In April 2012 the patient presented to the Gastroenterology Unit of the University of Padova Medical Centre. Biochemical testing confirmed abnormal cholestatic patterns that had been persisting since 2009 (γGT 229 IU/ml and ALP 201 IU/ml). At that time the patient showed: antimitochondrial antibody (AMA) positivity in immunofluorescence with a 1:160 titre, immunoblotting positivity for M2, 3E and anti pg 210, total serum cholesterol = 240 mg/dl (range ≤ 200 mg/dl), serum IgM = 7.31 g/L (range 0.4–2.38 g/L), normal transaminases, negative hepatitis B and C serum markers. The patient showed no symptom of cholestasis; the liver and spleen were not palpable. A liver biopsy, which was performed in May 2012, showed marked fibrosis of the portal tracts extending to the parenchyma, interface hepatitis, and a vanishing bile duct picture with the remaining ducts showing regressive alterations of cholangiocytes together with biliary metaplasia. The histological picture was compatible with stage III PBC. Ursodeoxycholic acid (UDCA), which was begun in April 2012 at 10 mg/Kg/day, was later increased to 15 mg/Kg/day; a partial reduction in γGT and ALP was recorded. In July 2012, the patient underwent Magnetic Resonance Imaging (MRI) cholangiography of the upper abdomen which revealed a marked reduction of the right segmental duct extended for 2.5 cm, with upstream dilatation, and pronounced stenosis of the biliary tree in the 5^th^ and 6^th^ hepatic segment considered characteristic of PSC. MRI typically shows multiple strictures and dilatations of the intrahepatic biliary tree in PSC patients. In our case, the coexistence of a liver biopsy compatible with PBC and MRI results typical of PSC permitted us to formulate a diagnosis of PBC-PSC overlap syndrome.

Given the absence of both the histopathological features of IgG4-related disease and abundant IgG4+ plasma cells in the liver biopsy material, the hypothesis of IgG4-associated cholangitis was excluded. Furthermore, there were no signs of synchronous involvement of other organs.

**Fig. 1:**
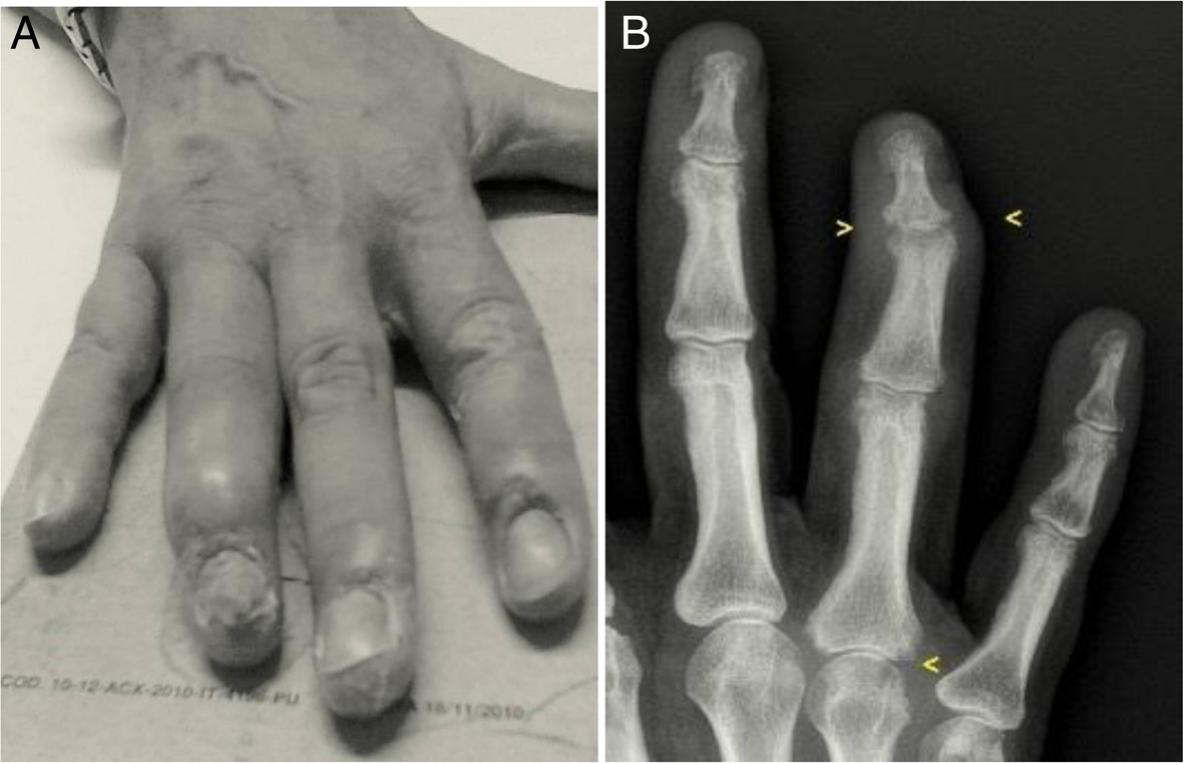
Right hand: arthritis of the fourth distal interphalangeal joint with severe nail psoriasis (**a**). Radiograph of the right hand: erosions in the fourth metacarpophalangeal and distal interphalangeal joints (**b**)

**Fig. 2:**
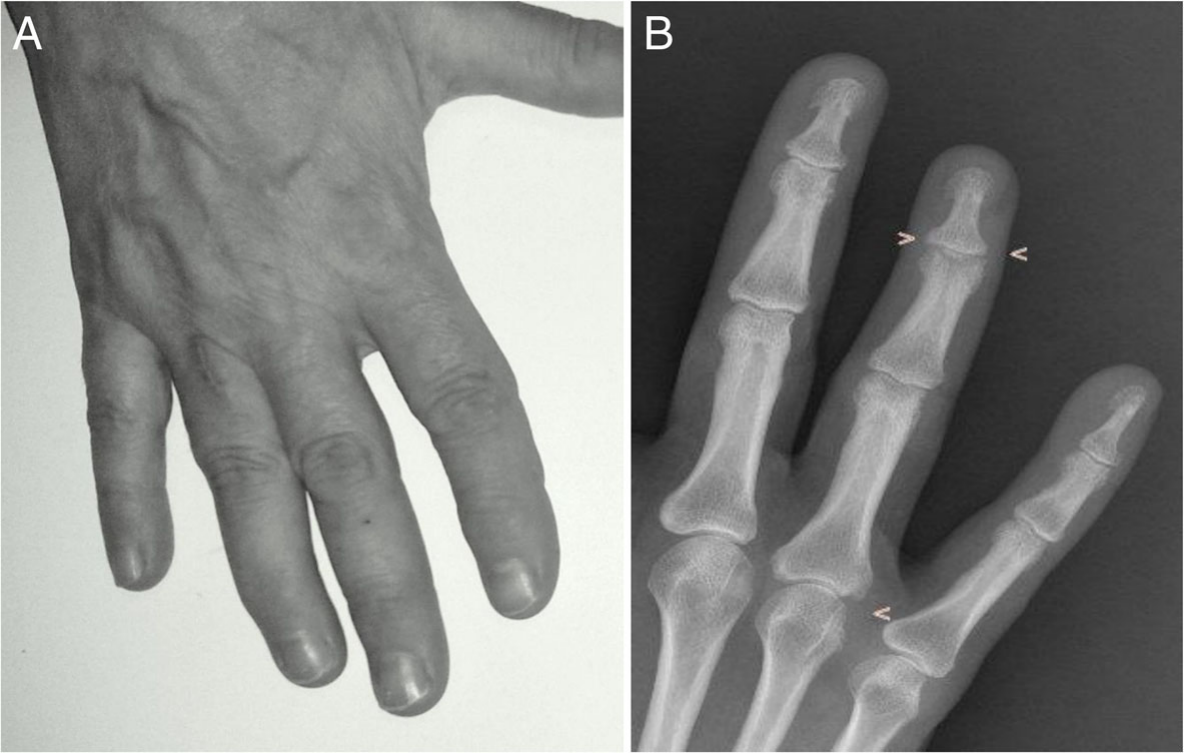
Right hand:swelling of the fourth distal interphalangeal joint was markedly improved, and nail appeared normal (**a**). Radiograph of the right hand: the bone erosion in the fourth distal interphalangeal joint seemed to have disappeared (**b**)

**Fig. 3:**
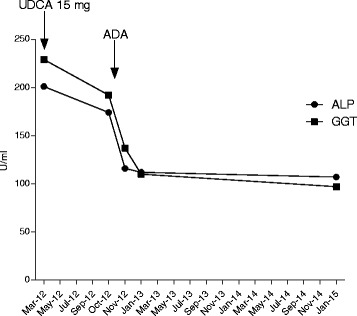
The cholestatic enzyme pattern during Adalimumab treatment. UDCA: ursodeoxycholic acid

In October 2012, arthritis of the fourth right DIP joint and severe nail psoriasis were two new symptoms that were added to the clinical picture (Fig. 1a). Hand X-rays showed new erosions in the fourth right MCF and DIP (Fig. 1b). Adalimumab treatment (40 mg fortnightly), which was begun at that time, led to notable relief of the pain and stiffness. Three months later the painful swellings of the second and fourth right DIP joints showed marked improvement, and the nail of the fourth right finger appeared normal (Fig. 2a). The patient’s Health Assessment Questionnaire (HAQ) disability index score fell from 0.750 to 0.375, the Disease Activity Score 28 (DAS28) fell from 4.25 to 3.18 and the Disease Activity in Psoriatic Arthritis (DAPSA) score fell from 17.37 to 4.29. Liver function tests improved, the ALP normalized, and the γGT and IgM improved (respectively falling to 124 IU/L and 5.35 g/L). Twelve months later, the HAQ continued to fall reaching 0.25, the DAS 28 fell to 1.80, and the DAPSA to 2.69. Hand X-rays showed that the bone erosion infourth right DIP joint seemed to have disappeared while the erosion at the base of the first phalanx persisted (Fig. 2b). Unfortunately, we do not have Computerized Tomography (CT) or MRI confirmation of these findings. Twenty-eight months later, the patient’s HAQ and DAS 28 values were stable, the DAPSA was 5.29 (low disease activity), the γGT and serum IgM had fallen even further (respectively reaching 98 IU/ml, and 4.93 g/L), and the ALP remained at normal levels (107 IU/ml) (Fig. 3). At present, the patient continues to receive Adalimumab and enjoys good health.

## Discussion

The current report describes for the first time a patient affected with PsA as well as with an overlapping PBC-PSC syndrome who was treated with Adalimumab which led to a clinical improvement in arthritis symptoms and lowered the indexes of cholestasis.

A recent study demonstrated a strong positive correlation of 44 non-human leukocyte antigen (HLA) loci for PSC that were also identified in seven immunological disorders including psoriasis [13]. In addition, susceptibility to PSC may be determined by polymorphisms of TNF genes [14]. The existence of TNF-α gene promoter variants that act as markers of both disease severity and response to treatment has been hypothesized for inflammatory arthropathies. The main TNF-α and TNF-α receptor polymorphisms also seem to be able to predict response to TNF-α blockers in PsA patients [15, 16]. As genetic factors play an important role in anti-TNF-α response in inflammatory arthropathies, it could be argued that they influence the effect of anti-TNF-α treatment in autoimmune colangiopathies or at least in some clinical phenotypes. It is also possible that different genetic combinations predispose patients to a particular clinical phenotype influencing response to treatment.

In the case outlined here in which PsA was associated with an overlapping PBC-PSC syndrome, the patient’s good response to TNF-α blockers seems to confirm the important role of TNF-α in the pathogenesis of these diseases. In addition, the fact that some TNF-α blockers are effective in different cholestatic autoimmune liver diseases in the presence of an inflammatory arthropathy is a particularly interesting, thought-provoking finding [9–12].

Adalimumab also proved to be effective in the case studied, even in connection to the radiographic alterations (bone erosions in the fourth right DIP) which seemed to disappear (Fig. 2b). It is nevertheless possible that the marked improvement in the patient’s bone condition was linked to the precocious therapeutic intervention that was implemented.

## Conclusion

Concomitant autoimmune diseases and/or immune-mediated disorders, which share genetic basis and possibly similar molecular pathways, have frequently been found in the same patient. As genetic factors play a vital role in the susceptibility to inflammatory arthropathies and in determining a drug’s effect, they may also be involved in the development of autoimmune colangiopathies and/or of some clinical phenotypes. A TNF-α polymorphism may explain both TNF-α’s pathogenic role in clinical subsets as well as the response to TNF-α blockers. In the case described here, the concomitant presence of PsA and the PBC-PSC overlap syndrome as well as the patient’s good response to TNF-α blockers all seem to confirm a common pathogenesis and point to the hypothesis that the use of anti-TNF-α drugs could be effective in one or more subsets of autoimmune colangiopathies. Notably, the clinical phenotype of the autoimmune colangiopathies that respond favorably to anti-TNF-α drugs seems to be the one in which inflammatory arthropathy is also present, as has been reported by our as well as other research groups.

Future studies will be able to verify the efficacy of TNF-α blockers in autoimmune cholangiopathies associated to inflammatory arthropathies and to identify the clinical subsets that are responsive to different anti-TNF-α agents.

## Abbreviations

ALP: Alkaline phosphatase
AMA: Antimitochondrial antibody
CRP: C-reactive protein
CT: Computerized tomography
DAPSA: Disease activity in psoriatic arthritis
DAS28: Disease activity score
DIP: Distal interphalangeal
ESR: Erythrocyte sedimentation rate
HAQ: Health Assessment Questionnaire
HLA: Human leukocyte antigen
IBD: Inflammatory bowel disease
MCP: Metacarpophalangeal
MRI: Magnetic resonance imaging
MTX: Methotrexate
PBC: Primary biliary cholangitis (formerly called primary biliary cirrhosis)
PsA: Psoriatic arthritis
PSC: Primary sclerosing cholangitis
RA: Rheumatoid arthritis
TNF-α: Tumour necrosis factor-α
UC: Ulcerative colitis
UDCA: Ursodeoxycholic acid
γGT: Gamma-glutamyltransferase
